# Longitudinal associations between cybervictimization, anger rumination, and cyberaggression

**DOI:** 10.1002/ab.21958

**Published:** 2021-03-03

**Authors:** Antonio Camacho, Rosario Ortega‐Ruiz, Eva M. Romera

**Affiliations:** ^1^ Universidad de Córdoba Cordoba Spain

**Keywords:** adolescents, anger rumination, cross‐lagged model, cyberbullying, longitudinal study

## Abstract

Adolescents' involvement in cyberbullying has been a growing public health concern for some time. Cybervictimization and cyberaggression are two phenomena that previous research has often shown to be associated. However, longitudinal research into these associations and also into potential risk factors for these phenomena is less common. Anger rumination is a proven risk factor for aggressive behavior, but the relationship between anger rumination and victimization is not clear. The present longitudinal study investigated the associations between cybervictimization, anger rumination and cyberbullying in a sample of 3017 adolescents (*M*_W1_ = 13.15; *SD* = 1.09; 49% girls) from 7th to 9th grade. The European Cyberbullying Intervention Project Questionnaire and the Anger Rumination Scale were administered in four waves with 6 months intervals over a total period of 18 months. The associations between the variables were analyzed with a cross‐lagged model. We found that: cybervictimization predicted anger rumination and cyberaggression; anger rumination was associated with later increases in both cybervictimization and cyberaggression: but involvement in cyberaggression predicted neither subsequent involvement in cybervictimization, nor in anger rumination. In addition, cybervictimization was found to mediate the association between anger rumination and cyberaggression. This study expands the understanding of the factors associated with cybervictimization and cyberaggression, and its results indicate that intervention programs should focus on boosting self‐control to decrease impulsive behavior and protocols to prevent and intervene in cyberbullying.

## 1 INTRODUCTION

Cyberbullying is often described as an intentional and aggressive behavior perpetrated by an individual or a group of individuals through the use of information and communication technologies (Smith et al., 2008). Previous research has tried to identify characteristics in adolescents associated with a heightened involvement in cybervictimization and cyberaggression. There is a wide body of evidence showing that cybervictimization and cyberaggression are associated (*r* = .21 to *r* = .80) (see meta‐analysis by Lozano‐Blasco et al., 2020). A meta‐analysis of the risk factors for cyberbullying found cybervictimization to be its strongest predictor (*r* = .51), but cyberaggression was not a risk factor for cybervictimization (Kowalski et al., 2014). There are different explanations for the association between victimization and aggression. The taxonomy of reasons (TOR) for involvement in aggressive behavior (Baumeister, 2001; Pinker, 2011) includes revenge (as planned behavior). Depending on their ability to cope with the negative emotions produced by cybervictimization, victims may experience a desire to take revenge and consequently get involved in reactive cyberaggression (Martins et al., 2019). Furthermore, aggression motivated by anger can also be impulsive, a form of self‐defense (Connor et al., 2019). Therefore, when victims feel threatened and attacked, especially if they feel the attack is unjustified, some may respond with anger and aggression (Fluck, 2017). Finally, negative emotions produced by online victimization (including anger) could weaken the ability to deal with social stress efficiently, which may lead to hostile processing of social information, which in turn can lead to cyberaggression (Ak et al., 2015; Marín‐López et al., 2019).

To design effective prevention programs, it is necessary not only to identify the risk and protective factors affecting involvement in cyberbullying, but also to understand the mechanisms underlying those relationships, and these remain largely unexplored (Romera et al., 2021). Anger rumination has received attention with regard to its association with (online) aggression, but its association with victimization remains less clear. This study examined the association of anger rumination, as a mechanism of internal state, with cybervictimization and cyberaggression. The possible mediating effects involving the association between variables were explored.

### 1.1 Anger rumination and cybervictimization

The response styles theory (Nolen‐Hoeksema, 1991) is often applied to study the negative effects of traumatic events, such as victimization. Responses to victimization are classified as emotion‐focused coping, aimed at minimizing distress by focusing on the affect related to the stressor, and problem‐focused coping, aimed at removing or, when unavoidable, minimizing the impact of the experience by focusing on the stressor (Lazarus & Folkman, 1984). While problem‐focused coping has been associated with prosocial and adaptive behavior, emotion‐focused coping has been linked to antisocial and aggressive behavior (Eisenberg et al., 2006). Within the response styles theory, emotion‐focused coping includes rumination, a cognitive process aimed at coping with negative experiences and feelings by repetitively and passively thinking about symptoms, causes and consequences (Nolen‐Hoeksema, 1991). The literature has differentiated trait rumination, the tendency to ruminate as a stable personality characteristic (Just & Alloy, 1997) and state rumination, referring to a focus on negative feelings and problems at a given point in time (Nolen‐Hoeksema & Morrow, 1993). The rumination referred to in the remainder of this article relates most closely to trait rumination. Previous studies about cyberbullying have found that anger was a common emotional response of adolescent victims (Ak et al., 2015; Ortega et al., 2012). In general, adolescents tend to regulate their emotions of anger, but the regulatory mechanisms do not always lead to an adaptive response. If an emotional state such as anger is retained over time, it can lead to rumination as a way to cope with the negative experience (Ray et al., 2008). Anger rumination can be understood as the tendency to focus on internal‐state‐related thoughts during an anger episode (Sukhodolsky et al., 2001). After such anger‐inducing incidents, some adolescents succeed in managing the situation, while others cannot stop thinking about the episode and how it came about (Li et al., 2019). Anger rumination has been shown to reduce the scope for adjusted response, such as reappraisal and problem solving (Lyubomirsky et al., 2015). Previous research has indeed shown that cybervictimization predicted rumination (Liu et al., 2020), but so far, the specific association with anger rumination remains largely unexplored. Only one descriptive study showed that adolescents and adults who were cybervictimized reported higher levels of anger rumination compared to those not involved in cyberbullying (Zsila et al., 2018).

Whether a reverse relationship also exists, that is, whether anger rumination predicts an increase in (cyber)victimization has not been studied yet, but this might be expected. Individuals who engage in anger rumination may be more inclined to focus on the negative feelings caused by the stressful event, rather than on addressing the problem. Previous research suggests that such emotion‐focused coping is associated with an increase in anxiety and depression (Izadpanah et al., 2017), which in turn are associated with cybervictimization (Wright & Wachs, 2019). Moreover, a deficit in self‐control, which is widely associated with anger rumination (White & Turner, 2014), is a proven risk factor for victimization online (Álvarez‐García et al., 2019). A meta‐analysis found that internalizing problems predict increased peer victimization during youth (Reijntjes et al., 2010). This may be explained by the fact that impulsive individuals tend not to consider the consequences of their actions when engaging in risky behaviors (Gottfredson & Hirschi, 1990). It should be noted that in Pratt et al. (2014) meta‐analysis the predictive effects of lacking self‐control on victimization proved greater with noncontact forms of victimization, such as in cyberspace. Based on the discussion above, it is expected that anger rumination is a risk factor for and predicts a subsequent increase in cybervictimization.

### 1.2 Anger rumination and cyberaggression

Emotion‐focused coping strategies activated by an anger‐raising event aim at managing the intensity of the anger experience, reducing angry thoughts and avoiding impulsive actions to prevent aggression (Denson et al., 2011). The multiple systems model of anger rumination (MSM) (Denson, 2013) has been applied in explaining how anger rumination might disturb the mechanisms of emotion regulation and thereby facilitate aggressive behavior. Through different levels of analysis (cognitive, neurobiological, affective, executive control, and behavioral, the MSM aims to understand why people engage in such cognitive processing after identifying an event as provocative. According to the model, the affective and neurobiological response, moderated by the cognitive response (e.g., through the mode of processing), influence executive control and aggressive behavior. The repetitive and passive thinking within anger rumination can overload cognitive processing (Denson, 2013) and consequently self‐control (White & Turner, 2014). Therefore, people with a high level of anger rumination and weak executive control have greater difficulties implementing the emotional regulation strategies that seek to decrease the arousal level, and thereby a greater propensity to behave aggressively, either impulsively or deliberately. Moreover, anger rumination can take the form of angry afterthoughts, thoughts of revenge, angry memories, and a focus on causes. Such processes tend to exacerbate and extend the anger emotion, and reconstructing the background of the threat or injury can create a willingness to engage in subsequent aggressive behavior (Denson et al., 2011).

On the empirical level, many studies have shown that anger rumination predicts higher levels of aggression (Quan et al., 2020; Salguero et al., 2020; Wang et al., 2019), but the opposite association, whether involvement in aggressive behavior predicts an increase in anger rumination, has not been analyzed. Moreover, with regard to cyberaggression specifically, as far as we know only one study—with a cross‐sectional design and using middle adolescents—has found an association with anger rumination (Yang et al., 2020). The scarcity of evidence relating to the association with cyberbullying perpetration is surprising, given cyberspace's abovementioned nature of anonymity (Barlett, 2015), which facilitates the possibility to take revenge with a lower probability of retaliation (Wright & Li, 2012). This warrants further investigation into anger rumination as a risk factor for cyberbullying perpetration, especially through longitudinal analysis.

### 1.3 The present study

Using the MSM as a theoretical foundation, the present panel study into the associations between cyberaggression, anger rumination and cybervictimization, therefore offers an important contribution to the existing literature about risk factors for cyberbullying among adolescents.

With the analysis of these associations, worth considering is also whether other factors, such as gender and age, influence these relationships. In a review of risk and protective factors for cyberbullying a definite relationship between gender and cyberbullying was not found, but the studies in this review that did report gender differences showed boys were more likely to be cyberbullying perpetrators, while girls were more likely to be cyberbullied (Kowalski et al., 2019). Regarding age, a recent study comparing pre‐adolescents and later adolescents found higher levels of involvement in both cybervictimization and cyberaggression in the older group (González‐Cabrera et al., 2019). With regard to anger rumination, higher levels have been found in girls than in boys (Zsila et al., 2019), and there is no existing information about differences between early and middle adolescents. Although there is some prior information to guide our expectations about level‐differences in these variables between boys and girls, and between early and middle adolescents, very little is known about the effect of gender and age, as moderating factors, on the associations between cybervictimization, anger rumination and cyberaggression. The limited studies that exist suggest gender does not influence the association between cybervictimization and cyberaggression (Chan et al., 2019), and between anger rumination and aggressive behavior (e.g., Guerra & White, 2017; White & Turner, 2014). While gender differences in the association between anger rumination and cybervictimization have not been explored yet.

In this study, we focus on adolescence because it is a life stage in which the presence of stressors increases (e.g., the unfair treatment from peers) (Lucas‐Thompson et al., 2018) and depending on attribution and coping style, a time when beneficial or maladaptive traits that affect later life are often adopted (Seiffge‐Krenke, 2013). The prevalence of cyberbullying increases during adolescence (González‐Cabrera et al., 2019), reaching its peak in the later phases of middle school (Kowalski et al., 2014).

Based on the abovementioned theoretical and empirical research, we formulated the following hypotheses: Adolescents who have been cybervictimized subsequently become more involved in cyber‐aggression (Hypothesis 1a); involvement in cyberaggression does not lead to a later increase or reduction in cybervictimization (Hypothesis 1b); cybervictimized adolescents show an increase in anger rumination (Hypothesis 2a), and anger rumination predicts an increase in cybervictimization (Hypothesis 2b); anger rumination predicts an increase in cyberaggression (Hypothesis 3a), but there is no reverse relationship (Hypothesis 3b); Finally, we expect that boys and girls do not differ in the associations between cybervictimization, anger rumination and cyberaggression (Hypothesis 4a), and that, due to the narrow age range of the study's participants (11–16 years, 7th–9th grade) there are no differences in the associations between early and middle adolescents (Hypothesis 4b).

The possible indirect effects were analyzed. We hypothesized that anger rumination would mediate the association between cybervictimization and cyberaggression (Hypothesis 5a). This is consistent with previous studies which found the mediating role of anger rumination as a risk mechanism of aggression with trait self‐control, trait anger and hostile attribution bias as predictors (Li et al., 2019; Quan et al., 2019; Wang et al., 2018). In a recent study, indirect effects of victimization and perpetration via anger rumination were found (Malamut & Salmivalli, 2021). In addition, as found in a longitudinal study the mediating role of victimization between depressive symptoms and violent behavior (Yu et al., 2018), we hypothesized that cybervictimization would mediate the association between anger rumination and cyberaggression (Hypothesis 5b).

## 2 METHODS

### 2.1 Participants

The participants were drawn from a large longitudinal study into personal and ecological developmental risks and protective factors during adolescence. The convenience sample comprised 3,017 adolescents (49% girls; 51% boys) between 11 and 16 years old, attending Grades 7–9, and included 115 classes from 13 middle schools in Southern Spain. In this study, we analyzed four waves of data collected during the years 2017–2019 at 6‐month intervals. At each data collection point the sample varied due to temporary absence or changes of school. Wave 1 (W1 hereafter) in November 2017 included 2790 adolescents (49% girls, *M*_age_ = 13.15, *SD* = 1.09, 92% participation rate); Wave 2 (W2) in May 2018 included 2553 (50% girls, *M*_age_ = 13.61, *SD* = 1.13, 85% participation rate); Wave 3 (W3) in November 2018 included adolescents 2362 (51% girls, *M*_age_ = 14.03, *SD* = 1.05, 78% participation rate); and Wave 4 (W4) in May 2019 included 2361 adolescents (50% girls, *M*_age_ = 14.55, *SD* = 1.06, 78% participation rate). Of the total sample, 59% participated in all four waves, 22% participated at three time points, 11% at two time points, and 7% took part at only one time point.

### 2.2 Measures

#### 2.2.1 Cyberbullying

We measured cybervictimization and cyberaggression using the European Cyberbullying Intervention Project Questionnaire (Del Rey et al., 2015). This scale has shown good validity and reliability in a Spanish population (Ortega‐Ruiz et al., 2016) and in cross‐cultural populations (Herrera‐López et al., 2017). The questionnaire includes 22 items that assess the frequency of cyberbullying behavior in two dimensions: 11 items assess cybervictimization (e.g., “Someone said nasty things about me to others either online or through text messages”) and 11 items assess cyberaggression (e.g., “I posted embarrassing videos or pictures of someone online”). The items were all answered on a 5‐point scale, ranging from 0 (*no*) to 4 (*yes, more than once a week*). Responses to the items were averaged within each dimension. Higher scores correspond to higher levels of cybervictimization and cyberaggression. The internal consistency of the scale in our study is presented in the Results section.

#### 2.2.2 Anger rumination

Anger rumination was measured with the Anger Rumination Scale (Sukhodolsky et al., 2001). This scale has shown good validity and reliability in Spanish populations (Uceda et al., 2016). The questionnaire includes 19 items (e.g., “When something makes me angry, I turn this matter over and over again in my mind,” “When someone provokes me, I keep wondering why this should have happened to me”). The items were answered on a 4‐point scale, ranging from 1 (almost never) to 4 (almost always). In line with previous studies using this scale, items were averaged to extract a global anger rumination score (Wang et al., 2019). Higher scores correspond to a higher level of anger rumination. The internal consistency of the scale in our study is presented in the Results section.

### 2.3 Procedure

Ethical approval was obtained from the research ethics committee of the corresponding author's institution. Before data collection, informed consent was obtained from government and school authorities, as well as from the participants' parents. The instruments were implemented in self‐report form in the classroom during regular school hours and included instructions on how to complete the questionnaire. Interviewers trained and experienced in psychological research supervised the data collection using standardized instructions. These included the assurance to participants that there were no right or wrong answers, that the data would be anonymous and treated confidentially, that participation was voluntary, and they could stop participating at any time. The researchers provided verbal reading support for those students with reading difficulties. The questionnaires were administered in paper‐and‐pencil format. Data from different waves was linked through a code composed of the first characters of the participants' given names and surnames, together with their dates of birth. On average, it took 30 min to answer the questionnaires.

### 2.4 Statistical analyses

Preliminary steps in the analysis included running descriptive statistics, correlations and independent *t*‐tests to explore gender (1 = boys; 2 = girls) and age (1 = early adolescents: 2 = middle adolescents) differences. The internal consistency of the scales was assessed with Cronbach's alpha. Longitudinal measurement invariance was analyzed to verify the consistency of the constructs over time (Little et al., 2013). Anger rumination was considered a global construct, while cybervictimization and cyberaggression were analyzed on the cyberbullying scale as two independent and correlated factors. It was done in a confirmatory factorial analysis by comparing three increasingly restrictive models. First, to test for configural invariance the model was estimated with the factor loadings and intercepts allowed to vary freely without restrictions. Then, metric invariance (weak) was analyzed after imposing equal factor loadings across time. Finally, scalar invariance (strong) was explored by imposing equal intercepts across time. Model fit of the three consecutive models was compared with determine the degree of invariance of the constructs. With regard to the comparison between models, ∆CFI < 0.01 and ∆RMSEA < 0.015 (Chen, 2007), they were considered to represent a statistically nonsignificant difference in model fit.

The associations between cybervictimization, anger rumination and cyberaggression were explored in a cross‐lagged model. This included the following paths: (a) autoregressive paths within the same variable over adjacent waves (e.g., anger rumination W1 → anger rumination W2); (b) cross‐lagged paths between different variables in adjacent waves (e.g., cybervictimization W1 → anger rumination W2); and (c) covariances between different variables measured at the same wave (e.g., anger rumination W1 ↔ cyberaggression W1), from W2 to W4, the covariances are based on the residual variances. To allow an efficient and systematic interpretation of the associations, we compared several models with decreasing constraints imposed on the estimation of abovementioned paths. Models were built in four steps: in Model 1 the cross‐lagged paths, autoregressive paths, and the residual covariances between the variables in the same wave were constrained to be equal over time (from W2 to W4); in Model 2 the residual covariances were freely estimated; in Model 3 the residual covariances and cross‐lagged paths were also freely estimated; and in addition, in Model 4 the residual covariances, cross‐lagged paths and autoregressive paths were freely estimated. The Scaled *χ* ^2^ Difference test (Satorra & Bentler, 2001) was considered to analyze whether the less constraint model fit better. In case of an improvement, the model with fewer constraints is retained, while no differences between the models are found, the model with higher constraints is used for further comparison with the next model. To analyze whether the longitudinal associations between cybervictimization, anger rumination, and cyberaggression differed between boys and girls, and early and middle adolescents, we ran multigroup analyses and verified results using Wald tests (Muthén & Muthén, 2017).

Analyses were conducted in M*plus* Version 8.4 (Muthén & Muthén, 2017). Models were estimated using the Maximum Likelihood Robust estimator (Satorra & Bentler, 2001) to account for non‐normality of the data. We reported standard fit indices, including the root mean square error of approximation (RMSEA), the comparative fit index (CFI), and the Tucker–Lewis index (TLI). RMSEA values < .08 and .05, and CFI and TLI values > .90 and .95 indicated acceptable and good model fit, respectively. To adjust the standard errors, we employed a “type = complex” sampling estimator, with classroom as a cluster variable, as adolescents were nested within previously defined groups. 5000 bootstrapping samples were conducted to estimate the confidence intervals for indirect effects through using INDIRECT model test in M*plus* to analyze the possible mediations between the variables from Time 1 to Time 4 (i.e., Time 1 → Time 2 → Time 3 → Time 4). Missing data character was explored though the Missing Completely at Random test (MCAR). Although Little's MCAR test provided a significant result (*p* < .001), correction of this result for sensitivity to sample size through the normed χ^2^ (χ^2^/*df* = 1.34) (Bollen, 1989) suggests data were missing at random (MAR). Full Information Maximum Likelihood (FIML) estimation was used to handle missing data, meaning all participants of the study were included in the analyses. By using all available data, FIML overcomes concerns associated with traditional missing data techniques, and provides an efficient estimation in longitudinal designs (Graham et al., 2001).

## 3 RESULTS

### 3.1 Preliminary steps

The means, standard deviations, and Cronbach's alpha of the main variables are displayed in Table 1, together with the independent *t*‐test results used to analyze gender and age differences. Girls scored higher on anger rumination than boys, while boys had higher cyberaggression scores in all waves and higher cybervictimization scores in W1. Two age groups were created to explore age differences, representing early (11–13 years) and middle (14–16 years) adolescence. Middle adolescents reported more cybervictimization, anger rumination, and cyberaggression than early adolescents. Following Cohen (1977), these effect sizes are considered small. Correlation analyses (see Table 2) showed that all variables were stable over time: *r* = .39 to *r* = .45 for cybervictimization; *r* = .47 to *r* = .62 for anger rumination; *r* = .24 to *r* = .36 for cyberaggression. All variables were significantly correlated cross‐sectionally and longitudinally in all waves. Respective coefficients of cross‐sectional and longitudinal correlation had the following ranges: for cybervictimization and anger rumination: *r* = .22 to *r* = .33; *r* = .12 to *r* = .24; for cybervictimization and cyberaggression: *r* = .64 to *r* = .72; *r* = .19 to *r* = .35; and for anger rumination and cyberaggression: *r* = .14 to *r* = .28; *r* = .10 to *r* = .16.

**Table 1:** Descriptive statistics and gender and age differences

				Gender		Age	
Variable	*M*	*SD*	*α*	*t*‐test	*d*	*t*‐test	*d*
1. Cybervictimization (T1)	0.24	0.45	.88	2.04*	0.09	−5.10***	0.21
2. Cybervictimization (T2)	0.21	0.38	.86	−0.98	–	−4.01***	0.18
3. Cybervictimization (T3)	0.19	0.39	.87	−0.26	–	−3.57***	0.16
4. Cybervictimization (T4)	0.20	0.38	.88	0.22	–	−2.72***	0.13
5. Anger rumination (T1)	2.03	0.67	.92	−3.94***	0.17	−5.67***	0.25
6. Anger rumination (T2)	2.08	0.72	.93	−6.11***	0.27	−2.80**	0.13
7. Anger rumination (T3)	2.04	0.71	.94	−5.71***	0.25	−2.69**	0.13
8. Anger rumination (T4)	2.11	0.74	.95	−6.40***	0.28	−1.97*	0.09
9. Cyberaggression (T1)	0.15	0.35	.87	5.36***	0.21	−7.16***	0.29
10. Cyberaggression (T2)	0.15	0.36	.90	2.79**	0.11	−3.43***	0.15
11. Cyberaggression (T3)	0.12	0.32	.90	2.35*	0.10	−3.61***	0.16
12. Cyberaggression (T4)	0.12	0.31	.90	2.02*	0.09	−2.27*	0.10

*Note*: The *t*‐test results show the differences of girls compared to boys and middle adolescents compared with early adolescents.

* *p* < .05.

** *p* < .01.

*** *p* < .001.

**Table 2:** Correlations between variables

Variables	1	2	3	4	5	6	7	8	9	10	11
1. Cybervictimization (T1)	–										
2. Cybervictimization (T2)	.39***	–									
3. Cybervictimization (T3)	.44***	.45***	–								
4. Cybervictimization (T4)	.37***	.41***	.45***	–							
5. Anger rumination (T1)	.33***	.24***	.22***	.20***	–						
6. Anger rumination (T2)	.21***	.27***	.20***	.20***	.56***	–					
7. Anger rumination (T3)	.20***	.18***	.26***	.19***	.51***	.61***	–				
8. Anger rumination (T4)	.14***	.12***	.19***	.22***	.47***	.55***	.62***	–			
9. Cyberaggression (T1)	.64***	.23***	.35***	.24***	.28***	.16***	.16***	.16***	–		
10. Cyberaggression (T2)	.24***	.70***	.25***	.22***	.12***	.20***	.14***	.13***	.24***	–	
11. Cyberaggression (T3)	.30***	.29***	.72***	.32***	.13***	.13***	.17***	.13***	.32***	.33***	–
12. Cyberaggression (T4)	.19***	.25***	.28***	.62***	.12***	.10***	.13***	.14***	.24***	.24***	.36***

*** *p* < .001.

To test for longitudinal measurement invariance of the scales, the factor loadings and intercepts were constrained to be equal over time in increasingly restrictive steps. The results indicated a good model fit for both scales (see Table 3). The nested model comparisons (Configural vs. Metric; Metric vs. Scalar) showed the increased constraints did not significantly affect model fit (∆CFI < 0.01 and ∆RMSEA < 0.015).

**Table 3:** Model fit: Testing for longitudinal measurement invariance

	Model fit indices				Model comparison		
Model tested	χ² (*df*) *p* value	CFI	TLI	RMSEA [90% CI]	∆ χ²_S‐B_ (*df*) *p* value	∆CFI	∆RMSEA
Cyberbullying							
Configural	4913.270 (3728)***	0.976	0.976	0.010 [0.010–0.011]	–	–	–
Metric	4952.604 (3783)***	0.977	0.976	0.010 [0.009–0.011]	91.75 (55)**	0.001	0.000
Scalar	5138.342 (3978)***	0.977	0.978	0.010 [0.009–0.011]	311.310 (195)***	0.000	0.000
Anger rumination							
Configural	7136.792 (2775)***	0.970	0.969	0.023 [0.022–0.024]	–	–	–
Metric	7198.826 (2826)***	0.970	0.970	0.023 [0.022–0.023]	127.149 (51)***	.0000	0.000
Scalar	7337.146 (2940)***	0.970	0.971	0.023 [0.022–0.023]	389.668 (114)***	0.000	0.000

Abbreviations: CFI, comparative fit index; CI, confidence interval; RMSEA, root mean square error of approximation; TLI, Tucker–Lewis index.

** *p* < .01.

*** *p* < .001.

### 3.2 Cross‐lagged model

We estimated and compared hierarchical cross‐lagged models to which constraints were introduced in a stepwise manner. Model 1 (with all paths constrained) had good model fit: *χ*² (57) = 165.090, *p* < .001; CFI = 0.979, TLI = 0.975; and RMSEA = 0.025, 90% CI [0.021–0.030]. After the covariances between variables in the same wave were allowed to vary over time, Model 2 did not reveal fit better than Model 1, consequently Model 1 was retained: (51) = 157.135, *p* < .001; CFI = 0.979, TLI = 0.973; and RMSEA = 0.026, 90% CI [0.022–0.031], ∆χ² (6) = 9.24, *p* > .05. Model 3, with additional unconstraint to the cross‐lagged paths, again showed good fit: χ² (40) = 136.671, *p* < .001; CFI = 0.981, TLI = 0.969; and RMSEA = 0.028, 90% CI [0.023–0.034]. Fit indices did not improve significantly from Model 1, ∆χ² (11) = 20.31, *p* > .05. Finally, Model 4, in which autoregressive paths were allowed to vary over time once more showed good fit: χ² (34) = 118.020, *p* < .001; CFI = 0.984, TLI = 0.968; and RMSEA = 0.029, 90% CI [0.023–0.035]. Model fit did not improve in comparison with Model 1, ∆χ² (23) = 33.1, *p* > .05. Given the lack of significant differences between the models, the Model 1 was used to analyze the associations between the variables.

**Figure 1:**
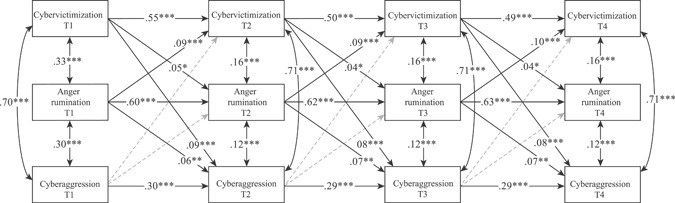
Cross‐lagged model. *Note*: The coefficients provided are the standardized values. Dashed arrows show nonsignificant paths. *p < .05, **p *<* .01, ***p < .001

The results of the cross‐lagged model are shown in Figure 1. The autoregressive paths were significant for all variables, as were all associations between variables within the same wave (W1) and the residual covariances (from W2 to W4). The cross‐lagged associations between different variables in adjacent waves indicate that: (a) cybervictimization predicted later anger rumination and cyberaggression, (b) anger rumination predicted later cybervictimization and cyberaggression; and (c) cyberaggression neither predicted later cybervictimization nor anger rumination. Sensitivity analyses were performed using multigroup modeling to test for gender and age differences: this implied constraining the cross‐lagged paths to be equal between: (a) boys and girls; and (b) early and middle adolescents. This did not lead to significant differences in any of the paths (*p*s > .05 for all Wald tests), indicating an absence of gender and age differences with regard to the associations. Based on the results found, the INDIRECT models were added to analyze the possible mediations between variables. The statistically significant mediated paths are presented in Table 4. The associations found in the cross‐lagged between the variables at two subsequent times are again confirmed through the indirect effects between Time 1 and Time 4. Furthermore, cybervictimization was found to mediate the association between anger rumination and cyberaggression.

**Table 4:** Significant indirect paths using bootstrap analysis

	*b* [95% CI]	*SE*	*t*	*p*
CV T1 → CV T2 → CV T3 → CV T4	0.130 [0.087–0.172]	0.02	55.59	<.001
CV T1 → CV T2 → CV T3 → AR T4	0.011 [0.001–0.021]	0.01	2.25	<.02
CV T1 → CV T2 → AR T3 → AR T4	0.015 [0.001–0.028]	0.01	2.15	<.05
CV T1 → AR T2 → AR T3 → AR T4	0.019 [0.000–0.038]	0.01	1.97	<.05
CV T1 → CV T2 → CV T3 → CA T4	0.022 [0.013–0.031]	0.01	4.69	<.001
CV T1 → CV T2 → CA T3 → CA T4	0.013 [0.006–0.020]	0.00	3.44	<.01
CV T1 → CA T2 → CA T3 → CA T4	0.008 [0.001–0.014]	0.00	2.21	<.05
AR T1 → AR T2 → AR T3 → AR T4	0.230 [0.200–0.261]	0.02	14.30	<.001
AR T1 → CV T2 → CV T3 → CA T4	0.003 [0.002–0.005]	0.00	3.72	<.001
AR T1 → CV T2 → CA T3 → CA T4	0.002 [0.001–0.003]	0.00	7.06	<.001
AR T1 → AR T2 → CV T3 → CA T4	0.004 [0.002–0.007]	0.00	3.22	<.01
AR T1 → AR T2 → AR T3 → CA T4	0.026 [0.005–0.046]	0.00	2.41	<.05
AR T1 → AR T2 → CA T3 → CA T4	0.012 [0.008–0.015]	0.00	6.16	<.001
AR T1 → CA T2 → CA T3 → CA T4	0.005 [0.003–0.007]	0.00	5.48	<.001
AR T1 → AR T2 → AR T3 → CV T4	0.035 [0.013–0.058]	0.01	3.08	<.01
AR T1 → AR T2 → CV T3 → CV T4	0.027 [0.015–0.040]	0.01	4.17	<.001
AR T1 → CV T2 → CV T3 → CV T4	0.021 [0.014–0.028]	0.00	5.73	<.001

Abbreviations: AR, anger rumination; CA, cyberaggression; CV, cybervictimization.

## 4 DISCUSSION

We looked at the longitudinal associations between anger rumination, cybervictimization and cyberaggression, as the possible mediation effects.

In previous research, cybervictimization and cyberaggression were strongly associated (Brewer & Kerslake, 2015), and our results support this: cybervictimization and cyberaggression would be positive and unidirectional. Our results supported that cybervictimization predicted further involvement in cyberaggression, not only through cross‐lagged effects but also through indirect effects between Time 1 and Time 4. However, a significant reverse relationship was not found. These findings are in line with Kowalski et al. (2014). Some adolescents may try to cope with the negative emotions caused by victimization through hostile reactions, either impulsively or deliberately. An additional explanation of the association is that the stress produced by victimization may result in an overly hostile interpretation of other social situations, which may then lead to cyberaggression that is not necessarily targeted at the original aggressor (Ak et al., 2015).

In our second hypothesis we formulated the expectation of a bidirectional association between anger rumination and cybervictimization. Via indirect and cross‐lagged effects, we indeed found that cybervictimization predicted a later increase in anger rumination, but perhaps more importantly as this had not been explored in the literature before, also found evidence of the reverse relationship: anger rumination predicted later victimization. With regard to the first of these associations, it was already known that some adolescents faced with cybervictimization will think repetitively about the experience and its causes, that is, that they turn to rumination (Liu et al., 2020). Furthermore, anger rumination also, or subsequently, predicts a greater likelihood of cybervictimization. This may be because adolescents who ruminate may be more vulnerable to impulsivity and consequently more likely to engage in risky behavior (Gottfredson & Hirschi, 1990), which in turn may upset or provoke others (e.g., teasing or joking with others), potentially leading to new episodes of victimization. The suggested explanation through impulsivity and risky behavior finds support in a result by Pratt et al. (2014) whose meta‐analysis showed that lower self‐control predicted subsequent cybervictimization. Our findings therefore highlight that the activation of anger rumination could be a strategy that leads to maladaptive behaviors, such as social anxiety or social maladjustment (Romera et al., 2016). An interesting element for future study related to this finding but not explored here, is how the level of social support and the role in/of the peer group (Romera et al., 2020) affects the association between anger rumination and cybervictimization. It can be imagined that an increase in risky behavior or a drop in self‐control is more likely to lead to further episodes of victimization for those adolescents with lower social support in their peer group.

In our third hypothesis we expected that anger rumination would predict a later increase in cyberaggression, but not the reverse. This pattern is indeed what we found through indirect and cross‐lagged effects, and is consistent with other studies (Yang et al., 2020). Our results highlight the importance of seeing anger rumination as a cognitive mechanism that increases the risk of adolescents turning to cyberaggression. According to the MSM, anger rumination may aggravate and sustain an internal state of aggressive thoughts and high arousal, and thereby lead cognitive processes to overload, which undermines appraisal and decision‐making abilities, and hence decreases the likelihood of self‐regulation and increases the likelihood of impulsive behavior (Denson, 2013). Anger rumination retrieves the offensive fact that caused the anger, leading to its intensification, which increases the probability of aggression. The anonymity and resulting reduced probability of retaliation offered by cyberspace lowers the bar for aggressive behavior. Cyberaggression offers adolescents an outlet to cope with the strained challenges they face on a daily basis, and this is an indication of the effects that their worries, perceptions and expectations have on the ability to process adverse experiences.

The expectations in the fourth hypothesis were met: the relationships between these variables did not differ between age groups or between boys and girls. This suggests that intervention programs aimed at anger rumination as a risk factor for cyberbullying should equally benefit boys and girls, as well as early and middle adolescents. While gender and age did not influence the associations, prevalence of rumination and bullying did differ between these groups: boys were more involved in cyberaggression, and girls more frequently reported rumination. Results for cybervictimization are ambivalent as there were differences at only one wave, with boys showing greater involvement than girls. With respect to age, middle adolescents showed greater involvement in all three study variables.

Finally, the present study extends the scope of the analysis beyond the reciprocal relationships between the variables, and furthermore the indirect effects revealed cybervictimization as a mediator, while anger rumination did not. In contrast to expectations and a recent study on face‐to‐face bullying (Malamut & Salmivalli, 2021), the association between cybervictimization and cyberaggression was not mediated by anger rumination (Hypothesis 5a). These considerations should remain cautious, as this study analyses rumination from a trait approach. Future research could clarify whether state anger rumination may act as a mediator, by activating such cognitive processes after the cybervictimization experience and subsequently lead to an increased probability of being involved in online aggression. On the other hand, despite extensive evidence of the association between anger rumination and aggressive behavior, the pathways linking them remain largely unknown. Our findings suggest that trait anger rumination is associated with aggression via victimization in the online context (Hypothesis 5b). This result extends beyond the current literature to understand why anger rumination is associated with cyberaggression. Adolescents at higher levels of anger rumination were at more risk of getting victimized online, and victimization experiences further increased the risk of aggression.

The results of this study should be interpreted in light of several limitations. First, although the sample of adolescents was large, we did not use a random sample. In future studies, our findings ideally should be replicated with a stratified random sample to ensure representativeness. Second, the novel study results need to be examined through other research techniques such as qualitative studies for a better understanding. This study only used self‐report instruments, which in future could be improved by the inclusion of multi‐informant data (e.g., from peers or family members). Finally, the students in this study all fell into a relatively narrow age range, which limits the ability to generalize our results to other age groups. Future studies may also want to assess whether the associations between cybervictimization, anger rumination and cyberaggression differ between those just starting to use technology as pre‐adolescents and later adolescents or emergent adults with more experience in cyberspace.

In spite of these limitations, this study offers a contribution to the growing body of research into factors associated with cybervictimization and cyberaggression among adolescents. As an empirical contribution, our findings show that anger rumination predicts an increase in later involvement in cybervictimization and cyberaggression, and that cybervictimization experiences and the anger they cause will in some adolescents lead to (higher levels) of anger rumination and cyberaggression. Finally, the study contributes to further insights into the association between anger rumination and cyberaggression by highlighting cybervictimization as a mediating mechanism between both.

The results of our study also have practical implications; they highlight the importance to develop cognitive strategies that improve self‐control to decrease impulsive and risky behavior and consequently victimization and aggression online. Denson et al. (2012) show how cognitive reappraisal strategies, such as dampening anger that is induced by flashbacks of anger can be successful. Their approach achieved adaptive processing of memories and promoted early reductions in anger experience through distraction strategies. Furthermore, other treatments such as mindfulness (Wright et al., 2009) and cognitive behavioral therapy (Querstret & Cropley, 2013) have proven effective in reducing anger rumination. In this line, previous studies highlight the main role that maladaptive cognitive emotion regulation strategies, like rumination and self‐blame, might play with regard to cyberbullying episodes (Rey et al., 2020) and how the promotion of forgiveness may decrease this association (Quintana‐Orts, Rey, 2018). In addition, it is particularly necessary that schools have evidence‐based protocols in place to prevent cyberbullying, and also intervention through restorative justice and reparation of damage (Del Rey et al., 2018; Williford et al., 2013). Our study supports that inclusion of such techniques and programs to prevent cyberbullying is likely to have beneficial effects.

## CONFLICT OF INTERESTS

The authors declare that there are no conflict of interests.

## Data Availability

The data that support the findings of this study are available on request from the corresponding author.
